# Prognosis of Early-Stage Hepatocellular Carcinoma: Comparison between Trans-Arterial Chemoembolization and Radiofrequency Ablation

**DOI:** 10.3390/cancers12092527

**Published:** 2020-09-05

**Authors:** Byung-Yoon Yun, Hye Won Lee, In Kyung Min, Seung Up Kim, Jun Yong Park, Do Young Kim, Sang Hoon Ahn, Beom Kyung Kim

**Affiliations:** 1 Department of Preventive Medicine, Yonsei University College of Medicine, Seoul 03722, Korea; yby3721@yuhs.ac; 2 Department of Internal Medicine, Yonsei University College of Medicine, Seoul 03722, Korea; lorry-lee@yuhs.ac (H.W.L.); ksukorea@yuhs.ac (S.U.K.); drpjy@yuhs.ac (J.Y.P.); dyk1025@yuhs.ac (D.Y.K.); ahnsh@yuhs.ac (S.H.A.); 3 Institute of Gastroenterology, Yonsei University College of Medicine, Seoul 03722, Korea; 4 Yonsei Liver Center, Severance Hospital, Seoul 03722, Korea; 5 Biostatistics Collaboration Unit, Department of Biomedical Systems Informatics, Yonsei University College of Medicine, Seoul 03722, Korea; iknice9@yuhs.ac

**Keywords:** radiofrequency ablation, chemoembolization, early-stage hepatocellular carcinoma, prognosis

## Abstract

**Simple Summary:**

Radiofrequency ablation (RFA) is a curative treatment for early-stage hepatocellular carcinoma (HCC) ineligible for surgery or liver transplantation. However, trans-arterial chemoembolization (TACE) might be an alternative when RFA is contraindicated due to structural problems. Among treatment-naive HCC patients fulfilling the Milan criteria who underwent RFA (*n* = 136) or TACE (*n* = 268), complete response (CR) and 5-year recurrence-free survival (RFS) rates were higher in the RFA group than in the TACE group (94.1% vs. 71.6% and 35.8% vs. 17.0%, respectively; both *p* < 0.001), whereas 5-year overall survival (OS) rates were not significantly different (65.5% vs. 72.3%, respectively; *p* = 0.100). After propensity-score matching, similar results were also reproduced. Hence, TACE could be an effective alternative to RFA in terms of OS rates. However, TACE should be confined only to RFA-difficult cases, given its lower CR and RFS rates and multi-disciplinary approaches are desirable in decision-making.

**Abstract:**

Radiofrequency ablation (RFA) is a curative treatment for early-stage hepatocellular carcinoma (HCC) ineligible for surgery or liver transplantation. However, trans-arterial chemoembolization (TACE) might be an alternative when RFA is contraindicated due to structural problems. Here, we aimed to compare their long-term outcomes. Treatment-naive HCC patients fulfilling the Milan criteria who underwent RFA (*n* = 136) or TACE (*n* = 268) were enrolled. Complete response (CR) and 5-year recurrence-free survival (RFS) rates were higher in the RFA group than in the TACE group (94.1% vs. 71.6% and 35.8% vs. 17.0%, respectively; both *p* < 0.001), whereas 5-year overall survival (OS) rates were not significantly different (65.5% vs. 72.3%, respectively; *p* = 0.100). Multivariate analysis showed that RFA was associated with better RFS (adjusted hazard ratio [aHR] 0.628; *p* = 0.001) than TACE, but not with better OS (aHR 1.325; *p* = 0.151). The most common 1st-line treatment after recurrence were TACE (*n* = 53), followed by RFA (*n* = 21) among the RFA group and TACE (*n* = 150), followed by RFA (*n* = 44) among the TACE group. After propensity-score matching, similar results were reproduced. Hence, TACE could be an effective alternative to RFA in terms of OS rates. However, TACE should be confined only to RFA-difficult cases, given its lower CR and RFS rates and multi-disciplinary approaches are desirable in decision-making.

## 1. Introduction

Currently, hepatocellular carcinoma (HCC) is a major health problem both in Korea and worldwide [1,2,3]. Hepatic resection or orthotopic liver transplantation (OLT) is the preferred curative treatment modality [4]. However, in the real-world practice, a substantial proportion of HCC patients are treated with various non-surgical options, primarily owing to tumor burden, the limited hepatic functional reserves, shortage of organ donors, high morbidities and mortalities associated with surgery, and patients’ refusals [5,6,7,8,9,10]. Among the non-surgical options, percutaneous radiofrequency ablation (RFA) is recommended as the first-line treatment in very-early-stage HCC (single tumor with a diameter < 2 cm) and as an alternative first-line treatment in early-stage HCC (single tumor size up to 5 cm, or up to three nodules with each having a maximal diameter < 3 cm), because it is less invasive and more tolerable than hepatic resection [11,12,13]. However, the rates of post-procedural recurrence (either local or distant) can be as high as 70% at 5 years, causing significant challenges with respect to long-term survival among patients treated with RFA [14].

Moreover, when tumors are located in the sub-capsular region, dome, or adjacent to intestinal loops or the bile duct, RFA is technically not feasible owing to higher risks of bleeding, perforation, or bile leakage [15]. In such cases, hepatic resection and OLT are also not feasible options. While selective trans-arterial chemoembolization (TACE) could be a viable alternative, it is primarily optimized as a palliative treatment for intermediate-to-advanced-stage HCC, despite several retrospective cohort studies indicating acceptable survival outcomes for patients with treatment-naïve early-stage HCC [16,17]. Moreover, the baseline tumor characteristics across previous studies on TACE were somewhat heterogeneous. Therefore, it remains unclear whether TACE and RFA provide a comparable outcome in early-stage HCC cases. 

Here, we aimed to compare the treatment efficacies and outcomes of TACE and RFA as the first-line modality for treatment-naïve patients with early-stage HCC who fulfilled the Milan criteria (single tumor size up to 5 cm or up to three nodules ≤ 3 cm; no vascular invasion; and no extrahepatic spread) by evaluating not only recurrence-free survival (RFS) rate and treatment response, but also the overall survival (OS) rate.

## 2. Materials and Methods 

### 2.1. Patients

Treatment-naïve patients with early-stage HCC who underwent RFA or TACE as an initial treatment at Severance Hospital, Seoul, Korea, between March 2005 and December 2016, were considered eligible for this study. The diagnosis of HCC was confirmed based on current practice guidelines of the American Association for the Study of Liver Diseases and the European Association for the Study of the Liver [4,18,19,20]. Liver biopsy was performed when diagnosis of HCC was unclear with clinical and radiological information. The inclusion criteria were as follows: age ≥ 19 years; presence of at least one uni-dimensional lesion measurable according to the modified response evaluation criteria in solid tumors (mRECIST) [21]; single tumor sized up to 5 cm or up to three nodules measuring ≤ 3 cm; Eastern Cooperative Oncology Group performance status of 0 to 2; platelet count ≥ 50×10^3^/µL; and serum alanine aminotransferase (ALT) levels <10 times the upper limit of normal. The exclusion criteria were as follows: history of anti-HCC treatment or portal or hepatic vein invasion; lymph node metastasis or extra-hepatic spread; Child–Pugh class C; any other uncontrolled co-morbidities or malignant neoplasms; and a prior OLT.

The patients provided written informed consent for the invasive procedures. The study protocol was in accordance with the ethical guidelines of the 1975 Declaration of Helsinki and was approved by the institutional review board of Severance Hospital (4-2018-0969).

### 2.2. Treatment Procedures and Follow-Up

RFA was performed based on the standard protocol of our institution. To assess the feasibility and applicability of RFA, a planning ultrasound was routinely performed. Then, the patients were treated percutaneously using an RFA device under ultrasound guidance. The tumor was ablated until ablation of the entire tumor was achieved. Immediately after RFA, complete ablation was confirmed using dynamic computed tomography (CT). 

TACE was also performed based on the standard protocol of our institution. The femoral artery was accessed via catheterization under ultrasound guidance. Hepatic angiography was performed to assess the vascular anatomy, tumor staining, and portal vein thrombosis. TACE was performed by infusion with a mixture of 5 mL iodized oil contrast medium (Lipiodol; Guerbet, Aulnay-sous-Bois, France) and 50 mg adriamycin, regardless of patients’ body weight, which was followed by embolization of the feeding arteries using gelatin sponge particles (Cutanplast; Mascia Bruneili S.p.A., Milano, Italy). The reduction of adriamycin dosage to 30 mg might be allowed according to the discretion of intervention radiologists, in case that the deterioration in the liver function after TACE was anticipated. The overall same procedure was performed for both single and multiple HCCs.

One month after RFA or TACE, laboratory tests including tumor markers, i.e., alpha-fetoprotein (AFP) and prothrombin induced by vitamin K absence-II (PIVKA-II), as well as imaging studies, i.e., dynamic CT or magnetic resonance imaging, were performed. Subsequently, the patients were followed-up every 3 months to monitor the recurrence after RFA or TACE. Treatment response was evaluated using mRECIST [21]. When the patient did not achieve complete response (CR), additional treatment was performed until the achievement of CR according to the physicians’ discretion.

### 2.3. Definitions and Evaluation of Data

The study endpoints were OS, RFS, and radiological response. OS was defined as the interval between initial treatment and death from any cause. RFS was defined as the period between initial treatment and radiologically confirmed recurrence, based on follow-up imaging data. Radiological response was evaluated according to mRECIST 4 weeks after RFA or TACE [21].

### 2.4. Statistical Analysis

The differences between the RFA and TACE groups were compared using the independent t-test for continuous data, and the chi-square test or Fisher’s exact test for categorical data. OS and RFS were estimated using the Kaplan–Meier curve and compared using the log-rank test. To identify the factors associated with RFS or OS, a Cox regression model was used to calculate the hazard ratio (HR) and 95% confidence interval (CI) for each variable in the univariate and multivariate analysis.

Furthermore, to reduce the effect of selection bias and potential confounders between the RFA and TACE groups, the propensity score (PS) was calculated using logistic regression, and PS-matching was performed at a 1:1 ratio using age, male sex, etiology, presence of liver cirrhosis, platelet count, Child-Pugh class, tumor size (expressed as a sum of each tumor diameter), single tumor, AFP, and PIVKA-II as adjusted variables.

A *p*-value less than 0.05 was considered significant. All statistical analyses were performed using R, version 3.6.0 (the R Foundation for Statistical Computine, Vienna, Austria) with ‘survival’ package, and SAS (version 9.4, SAS Inc., Cary, NC, USA).

## 3. Results

### 3.1. Patients’ Baseline Characteristics

A total of 404 patients, including 268 in the TACE group and 136 in the RFA group, were finally enrolled. Patients’ baseline characteristics are summarized in Table 1. The overall median age was 69.3 years, with a predominance of men (73.0%). The most common etiology of HCC was hepatitis B virus infection (70.3%), followed by hepatitis C virus infection (14.6%). The non-viral etiologies were as follows; alcohol (*n* = 28), non-alcoholic steatohepatitis (*n* = 13), autoimmune liver disease (*n* = 1), and others (*n* = 19). Twenty-two patients were diagnosed as HCC based upon pathological data and their histological differentiation was as follows: Edmondson grade I (*n* = 16), grade II (*n* = 5), and grade 3 (*n* = 1).

Most patients had a single tumor (77.5%), liver cirrhosis (77.7%), and Child-Pugh class A (89.9%). On comparing the baseline clinical characteristics between the two groups, the TACE group was found to have a larger average tumor size (expressed as a sum of each tumor diameter) (2.83 ± 1.11 vs. 1.98 ± 0.69 cm, respectively; *p* < 0.001) and lower proportion of single tumors than the RFA group (68.7% vs. 94.9%, respectively; *p* < 0.001). However, there were no significant differences in other variables between the two groups. 

### 3.2. Radiological and Biological Treatment Response for the Entire Cohort

Table 2 shows the radiological response, which was assessed through mRECIST 4 weeks after treatment [18]. Figures S1 and S2 showed the representative images before RFA or TACE and CR cases after treatment. Evidently, the RFA group had a higher proportion of CR (94.1% vs. 71.7%) and objective response (sum of CR and partial response) (97.8% vs. 87.7%) than the TACE group (both *p* < 0.001).

Among 388 patients where both AFP and PIVKA-II levels 4 weeks after treatment are available, patients with an objective response are more likely to have an AFP response (defined as ≥ 50% reduction from baseline) than those without (36.2% vs. 14.7%, respectively; *p* = 0.013). However, in terms of PIVKA-II response (also defined as ≥ 50% reduction from baseline), there exists only a trend toward the higher biological response in those with objective response, compared to those without (30.2% vs. 20.6%, respectively; *p* = 0.239) (Table S1).

### 3.3. Comparison of Survival Outcomes between the Two Groups for the Entire Cohort

**Figure 1:**
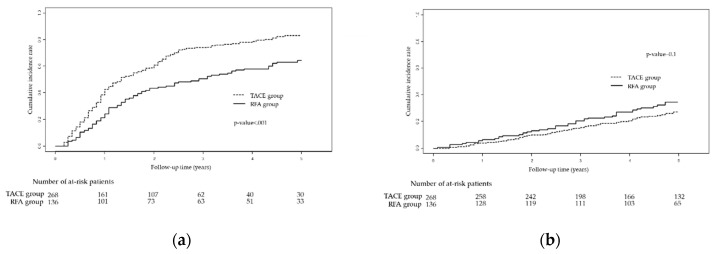
Kaplan-Meier curves showing (**a**) recurrence-free survival and (**b**) overall survival for TACE vs. RFA groups based on the entire cohort. Abbreviations: TACE, trans-arterial chemoembolization; RFA, radiofrequency ablation.

The RFS in the two groups was significantly different (Figure 1a; *p* < 0.001). The RFA group showed a significantly better 5-year RFS rate (35.8% vs. 17.0%; HR 0.560, 95% CI 0.432–0.726; *p* < 0.001) than the TACE group; the cumulative probabilities of recurrence at 1, 3, and 5 years in the TACE group were 42.8%, 73.9%, and 83.0%, respectively. In contrast, those in the RFA group were 24.4%, 50.7%, and 64.2%, respectively (Figure 1a).

Conversely, OS was not significantly different between the two groups (Figure 1b; *p* = 0.100). The 5-year OS rate in the RFA group was 65.5%, compared with 72.3% in the TACE group (HR 1.330, 95% CI 0.910–1.943; *p* = 0.141); the cumulative probabilities of death at 1, 3, and 5 years in the TACE group were 4.1%, 15.6%, and 27.7%, respectively. In contrast, those in the RFA group were 6.62%, 20.6%, and 34.5%, respectively (Figure 1b). 

### 3.4. Estimation of Predictors for Recurrence and Death in the Entire Cohort

Univariate and multivariate Cox regression analyses were performed to determine independent prognostic factors for recurrence and death. Univariate predictors were included in the multivariate analysis to assess the independent associations between the variables and clinical outcomes. RFA (vs. TACE) was associated with a lower probability of recurrence (adjusted HR 0.628, 95% CI 0.473–0.834, *p* = 0.001]); however, other variables did not have independent associations with recurrence (Table S2). In contrast, age, liver cirrhosis, and Child-Pugh class B were significant univariate predictors for death (all *p* < 0.05). After adjusting for these variables, we found that RFA (vs. TACE) was not associated with death (adjusted HR 1.325, 95% CI 0.902–1.948; *p* = 0.151). Finally, age (adjusted HR 1.042, 95% CI 1.019–1.067; *p* < 0.001) and Child-Pugh class B (adjusted HR 2.167, 95% CI 1.291–3.636; *p* = 0.003) were revealed to be independent predictors of death based on multivariate analysis (Table S3).

### 3.5. Subsequent Treatments after Recurrence

The first-line treatment modalities against the recurred lesion were as follows; TACE (*n* = 53), followed by RFA (*n* = 21), best supportive care (*n* = 3), surgical resection or OLT (*n* = 2), and systemic chemotherapy (*n* = 1) among the RFA group and TACE (*n* = 150), followed by RFA (*n* = 44), best supportive care (*n* = 8), surgical resection or OLT (*n* = 5), liver-directed concurrent chemoradiotherapy (*n* = 2), systemic chemotherapy (*n* = 2), and external beam radiotherapy (*n* = 1) among the TACE group. 

During the follow-up, 16 patients among the RFA group experienced extra-hepatic spread (i.e., M1) and/or lymph node metastasis (i.e., N1), while 47 among the TACE group did. 

Furthermore, as their first-line systemic chemotherapy during the follow-up, 13 and two patients among the RFA group were treated with sorafenib and lenvatinib, whereas 33, one, and one patients among the TACE group were treated with sorafenib, lenvatinib, and cytotoxic chemotherapy, respectively.

### 3.6. Analyses in the PS-Matched Cohort

Among a total of 404 patients, PS-matching provided 122 pairs. The baseline clinical characteristics of the patients after PS-matching are summarized in Table 3, which also shows the acceptable balance between the TACE and RFA groups (all *p* > 0.05). Table 4 shows the radiological response in the PS-matched cohort according to the treatment modality used (TACE vs. RFA). We found that the RFA group had a higher proportion of CR (94.3% vs. 73.8%) and objective response (97.6% vs. 85.3%) than the TACE group (both *p* < 0.001), even after PS-matching.

**Figure 2:**
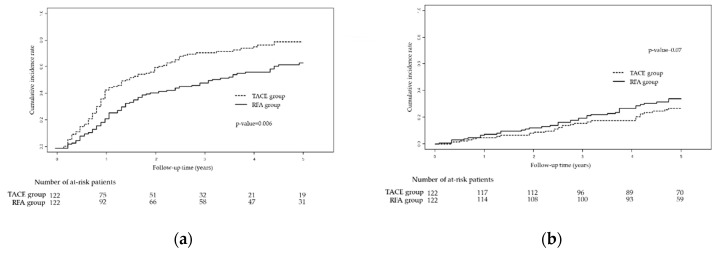
Kaplan-Meier curves showing (**a**) recurrence-free survival and (**b**) overall survival in the TACE vs. RFA groups after adjustment through propensity score-matching. Abbreviations: TACE, trans-arterial chemoembolization; RFA, radiofrequency ablation.

Similar results were also reproduced for recurrence; the RFA group had a better 5-year RFS rate than the TACE group (Figure 2a; 36.0% vs. 20.1%; *p* = 0.006). The cumulative probabilities of recurrence at 1, 3, and 5 years in the TACE group were 43.9%, 71.7%, and 79.9%, respectively, whereas those in the RFA group were 22.2%, 49.1%, and 64.0%, respectively (Figure 2a). 

In terms of deaths, there was no significant difference in the 5-year OS rate between the TACE and RFA groups (Figure 2b; 73.1% vs. 65.9%; *p* = 0.07). The cumulative probabilities of death at 1, 3, and 5 years in the TACE group were 4.9%, 15.7%, and 26.9%, respectively, whereas those in the RFA group were 7.4%, 19.7%, and 34.1%, respectively (Figure 2b).

## 4. Discussion

For patients with unresectable HCC who fulfill the Milan criteria, OLT and RFA are generally accepted as the principal choices with a curative aim. Among these, OLT is largely limited by the donor organ shortage, especially in the Korea, where living donor liver transplantation currently accounts for more than 80% of cases. Although RFA is considered the treatment of choice for early-stage HCC in the real-word practice, a substantial number of HCC patients still undergo TACE as the first-line treatment for tumor control and survival prolongation [22]. Here, we assessed the survival benefit among early-stage HCC patients who underwent TACE instead of RFA for technical reasons. This study has several strengths. First, it had a relatively large sample size (*n* = 404), in comparison with that in previous studies [16,23,24], as well as a sufficient number of events during the long-term follow-up, which provided significant statistical power. Second, our study results were consistently reproduced through multivariate Cox regression and PS-matching analyses. 

We found no significant difference between the TACE and RFA groups with regard to the 5-year OS rate in early-stage HCC patients. In other words, when RFA is not technically feasible, patients with early-stage HCC, including those with a single tumor, can achieve the same therapeutic benefit in terms of OS through TACE [25], although, as expected, the TACE group had a poorer CR and 5-year RFS rate than the RFA group. 

The inferior CR and 5-year RFS rates with TACE are consistent with theoretical perspectives; RFA is performed through thermal ablation of the tumor itself, whereas TACE is performed by injecting chemotherapeutic agents in tumor feeding vessels. Even though arteries are blocked by TACE, tumors attempt to develop new vessels through angiogenesis to receive blood supply from the hepatic portal vein [26,27]. Moreover, well-differentiated parts of HCC tumors are often fed by portal veins [28], also leading to a relatively lower local control rate with TACE. This discrepancy between analyses of death and recurrence according to the treatment modality might, in part, be explained by several hypotheses. First, it is generally recognized that most patients with HCC encounter multiple treatment failure events, not only at the treatment site, but also at other intrahepatic sites, owing to de novo carcinogenesis. Therefore, subsequent treatments, such as repeated RFA, TACE, external beam radiation therapy, or other systemic therapy, are commonly performed after initial single loco-regional treatment (LRT). Second, the survival of patients with HCC is not only strongly associated with tumor factors, but also with the severity of underlying liver dysfunction, evidenced by the observation that age and Child-Pugh class were independent predictors of OS in the present study. Consequently, the higher CR rate and lower recurrence rate achieved with RFA may not directly translate into better long-term OS benefits. 

Nevertheless, TACE cannot be equated with RFA while choosing an optimal treatment strategy for early-stage HCC. Our results indicated that RFA is more advantageous from the perspectives of medical expenses and quality of life, since it has better CR and RFS rates than TACE. Furthermore, for patients with anatomical problems, i.e., presence of bland portal vein thrombosis or arterio-portal shunt, and with Child-Pugh class B, TACE, as an alternative treatment modality to RFA, might have a limited therapeutic performance, primarily owing to risk of deterioration in the liver function after treatment. Therefore, TACE should be confined only to RFA-difficult cases. A well-designed randomized controlled trial would be ideal to obtain robust evidence on the therapeutic benefits of TACE and RFA. However, this is not completely feasible, primarily owing to the current concept that TACE is essentially one of the palliative treatment options for large and/or multinodular HCCs and the resultant ethical concerns. In the same context, in our study, the RFA group tended to have a smaller tumor size and a higher proportion of single tumors, indicating that most hepatologists prefer RFA to TACE for smaller and solitary tumors, consistent with the requisite guidelines [4]. 

Additionally, several studies have implied that a radiologic response to LRT is an important factor for predicting the patients’ long-term survival outcome [29,30]. Particularly, for those treated with TACE, the achievement of CR after the first TACE is of paramount significance for maintaining long-term OS [31]. As the effect of the initial TACE is the most important for improving OS, and multiple TACE sessions may have unfavorable effects on liver function, during the initial TACE procedure, maximizing total tumor necrosis might be preferable in practice, as far as permitted by hepatic function [32]. Furthermore, considering that angiogenic factors associated with poor prognosis, such as serum vascular endothelial growth factor and hypoxia-inducible factor-1 alpha, tend to increase in suboptimal responders after TACE [33], further efforts to achieve CR would also be required. Therefore, combination therapy with RFA, external beam radiotherapy, or adjuvant targeted therapy along with an initial cycle of TACE, if necessary, could also be considered to achieve complete necrosis. Actually, substantial numbers of patients among the RFA group were subsequently treated using TACE for recurred HCC and those in the TACE group were also treated using subsequent RFA. It indicates that multi-disciplinary approaches for the recurred lesion, as well as an initial treatment modality, should be important in RFA-difficult patients in order to prolong their survival outcomes.

There are several limitations to this study. First, it was a retrospective study conducted in a single institute, which may introduce selection bias, particularly in terms of patients’ demographic characteristics and treatment allocation. However, to overcome this, we performed various statistical adjustments, confirming that similar patterns of results were consistently reproduced. A multi-center, prospective randomized study is needed, although such a study is unlikely to be conducted in the near future. Therefore, this observational study has considerable scientific value. Second, recently, LRTs other than TACE have become popular as an alternative to RFA. For example, stereotactic body radiation therapy might provide similar or better local control rates than RFA in some cases [34], and microwave ablation could be more advantageous in terms of increased ablation volumes, sustained tissue temperatures, shorter duration of therapy, and susceptibility to the heat sink effect for HCCs located adjacent to vascular structures [35]. Thus, to select personalized treatment options, further studies are required to assess the short- and long-term efficacy of each modality and risk factors for treatment failure. Finally, our results might not be generalizable to the full spectrum of HCC patients, as chronic HBV infection was the most predominant etiology among our study population.

## 5. Conclusions

In conclusion, TACE could be an effective alternative to RFA in terms of the 5-year OS rate for early-stage HCC, provided that appropriate subsequent treatments are administered. However, TACE should be confined only to RFA-difficult cases, given its lower CR and RFS rates and risk of deterioration in the liver function after treatment, as compared with RFA.

## Figures and Tables

**Table 1:** Comparison of baseline clinical characteristics between the two groups before adjustment.

Variables	TACE Group (*n* = 268)	RFA Group (*n* = 136)	*p*-Value
Age, years	69.3 ± 9.0	69.1 ± 10.1	0.840
Male	193 (72.0%)	102 (75.0%)	0.523
Etiology			0.153
HBV	195 (72.8%)	89 (65.4%)	
HCV	39 (14.6%)	20 (14.7%)	
Non-B, Non-C	34 (12.6%)	27 (19.9%)	
Liver cirrhosis	210 (78.4%)	104 (76.5%)	0.667
Child-Pugh class			0.444
A	243 (90.7%)	120 (88.2%)	
B	25 (9.3%)	16 (11.8%)	
Platelet count, ×10^3^/uL	117 ± 50.9	128 ± 59.9	0.080
Tumor size, cm	2.83 ± 1.11	1.98 ± 0.69	<0.0001
Single tumor	184 (68.7%)	129 (94.9%)	<0.001
AFP, ng/mL	181.9 ± 625.3	159.5 ± 494.4	0.695
PIVKA-II			0.056
<1000 mAU/mL	260 (97.01%)	136 (100%)	
≥1000 mAU/mL	8 (2.99%)	0 (0%)	

Abbreviations: TACE, trans-arterial chemoembolization; RFA, radiofrequency ablation; HBV, hepatitis B virus; HCV, hepatitis C virus; AFP, alpha-fetoprotein; PIVKA-II, prothrombin induced by vitamin K absence-II.

**Table 2:** Radiological response according to the treatment modality in the entire population.

Radiological Response	TACE Group (*n* = 268)	RFA Group (*n* = 136)	*p*-Value
			<0.001
Complete response	192 (71.64%)	128 (94.12%)	
Partial response	43 (16.04%)	5 (3.68%)	
Stable disease	29 (10.82%)	2 (1.47%)	
Progressive disease	4 (1.49%)	1 (0.74%)	

Abbreviations: TACE, trans-arterial chemoembolization; RFA, radiofrequency ablation.

**Table 3:** Comparison of baseline clinical characteristics between the two groups after adjustment using propensity score (PS)-matching.

Variables	TACE Group (*n* = 122)	RFA Group (*n* = 122)	*p*-Value
Age, years	68.8 ± 9.1	69.0 ± 9.8	0.840
Male	85 (69.7%)	90 (73.8%)	0.435
Etiology			0.599
HBV	82 (67.2%)	82 (67.2%)	
HCV	19 (15.6%)	17 (13.9%)	
Non-B, Non-C	21 (17.2%)	23 (18.9%)	
Liver cirrhosis	95 (77.9%)	95 (77.9%)	>0.999
Child-Pugh class			0.706
A	109 (89.3%)	107 (87.7%)	
B	13 (10.7%)	15 (12.3%)	
Platelet count, ×10^3^/uL	122 ± 54.2	122.4 ± 58.7	0.922
Tumor size, cm	2.03 ± 0.75	2.03 ± 0.70	0.9943
Single tumor	117 (95.9%)	115 (94.3%)	0.480
AFP, ng/mL	139.1 ± 471.8	155.5 ± 484.0	0.780
PIVKA-II			>0.9999
<1000 mAU/mL	122 (100.00%)	122 (100.00%)	
≥1000 mAU/mL	0 (0.00%)	0 (0.00%)	

Abbreviations: PS, propensity score; TACE, trans-arterial chemoembolization; RFA, radiofrequency ablation; HBV, hepatitis B virus; HCV, hepatitis C virus; AFP, alpha-fetoprotein; PIVKA-II, prothrombin induced by vitamin K absence-II.

**Table 4:** Radiological response according to the treatment modality after adjustment through PS-matching.

Radiological Response	TACE Group (*n* = 122)	RFA Group (*n* = 122)	*p*-Value
			0.001
Complete response	90 (73.77%)	115 (94.26%)	
Partial response	14 (11.48%)	4 (3.28%)	
Stable disease	16 (13.11%)	2 (1.64%)	
Progressive disease	2 (1.64%)	1 (0.82%)	

Abbreviations: PS, propensity score; TACE, trans-arterial chemoembolization; RFA, radiofrequency ablation.

## Supplementary Materials

The following are available online at https://www.mdpi.com/2072-6694/12/9/2527/s1; Table S1: Association between radiological and biological responses (*n* = 388), Table S2: Predictors of recurrence, Table S3: Predictors of death, Figure S1: Representative images where the red and yellow arrows indicate HCC before RFA (A) and CR status after RFA (B), respectively, Figure S2: Representative images where the red and yellow arrows indicate HCC before TACE (A) and CR status after TACE (B), respectively.
