# Supplementary material for: Prognosis of Early-Stage Hepatocellular Carcinoma: Comparison between Trans-Arterial Chemoembolization and Radiofrequency Ablation
Source: Cancers (Basel). 2020 Sep 5;12(9):2527. doi: 10.3390/cancers12092527 (PMC7565721; doi:10.3390/cancers12092527)
Supplement: Supplementary file 1 [file cancers-12-02527-s001.pdf]

Supplementary materials

Prognosis of Early-Stage Hepatocellular Carcinoma: Comparison between Trans-Arterial Chemoembolization and Radiofrequency Ablation

Byung-Yoon Yun, Hye Won Lee, In Kyung Min, Seung Up Kim, Jun Yong Park, Do Young Kim, Sang Hoon Ahn and Beom Kyung Kim

Table S1. Association between radiological and biological responses (n=388).

		Radiological response				
		**Objective response**	**Non-response**		**Objective response**	**Non-response**
**Biological response**	**AFP response**	128 (36.2%)	5 (14.7%)	PIVKA-II response	107 (30.2%)	7 (20.6%)
	**AFP non-response**	226 (63.8%)	29 (85.3%)	PIVKA-II non-response	247 (69.8%)	27 (79.4%)

Abbreviations: AFP, alpha-fetoprotein; PIVKA-II, prothrombin induced by vitamin K absence-II

Table S2. Predictors for recurrence

**Variables**	**Univariate analysis**		**Multivariate analysis**	
	**HR (95% CI)**	**p-value**	**Adjusted HR (95% CI)**	**p-value**
Age, years	1.013 (1.001-1.026)	0.034	1.009 (0.995-1.022)	0.219
Female	1.099 (0.851-1.418)	0.469		
Etiology				
HBV	1	Ref	1	Ref
HCV	1.46 (1.073-1.986)	0.016	1.368 (0.980-1.911)	0.066
Non-B, Non-C	0.899 (0.634-1.277)	0.552	0.917 (0.639-1.315)	0.637
Liver cirrhosis	0.956 (0.729-1.252)	0.742		
Child-Pugh class B (vs.A)	1.091 (0.743-1.601)	0.657		
Platelet count	0.998 (0.996-1.000)	0.067		
Tumor size	1.212 (1.092-1.345)	<0.001	1.06 (0.925-1.215)	0.403
Tumor number > 1	1.547 (1.192-2.006)	0.001	1.211 (0.876-1.674)	0.248
AFP, ng/mL	1 (0.999-1.000)	0.902		

PIVKA-II, mAU/mL	1 (0.999-1.000)	0.289		
RFA (vs. TACE)	0.560 (0.432-0.726)	<0.001	0.628 (0.473-0.834)	0.001

Abbreviations: HR, hazard ratio; CI, confidence interval; HBV, hepatitis B virus; Ref, reference; HCV, hepatitis C virus; AFP, alpha-fetoprotein; PIVKA-II, prothrombin induced by vitamin K absence-II; RFA, radiofrequency ablation; TACE, trans-arterial chemoembolization

Table S3. Predictors for death

Variables	Univariate analysis		Multivariate analysis	
	HR (95% CI)	p-value	Adjusted HR (95% CI)	p-value
Age, years	1.045(1.023-1.067)	<0.001	1.042(1.019-1.067)	<0.001
Female	1.185(0.792-1.775)	0.409		
Etiology				
HBV	1	Ref	1	Ref
HCV	1.814(1.119-2.940)	0.016	1.092(0.639-1.865)	0.747
Non-B, Non-C	1.93(1.200-3.104)	0.007	1.410(0.853-2.330)	0.181
Liver cirrhosis	1.676(1.011-2.776)	0.045	1.498(0.901-2.492)	0.404
Child-Pugh class B (vs.A)	2.180(1.330-3.573)	0.002	2.167(1.291-3.636)	0.003
Platelet count	0.997(0.994-1.001)	0.205		
AFP, ng/mL	1(0.999-1.000)	0.623		
PIVKA-II, mAU/mL	1(0.997-1.000)	0.564		
Tumor size	1.094(0.922-1.298)	0.304		
Tumor number > 1	0.806(0.501-1.296)	0.374		
RFA (vs. TACE)	1.33(0.910-1.943)	0.141	1.325(0.902-1.948)	0.151

Abbreviations: HR, hazard ratio; CI, confidence interval; HBV, hepatitis B virus; Ref, reference; HCV, hepatitis C virus; AFP, alpha-fetoprotein; PIVKA-II, prothrombin induced by vitamin K absence-II; RFA, radiofrequency ablation; TACE, trans-arterial chemoembolization

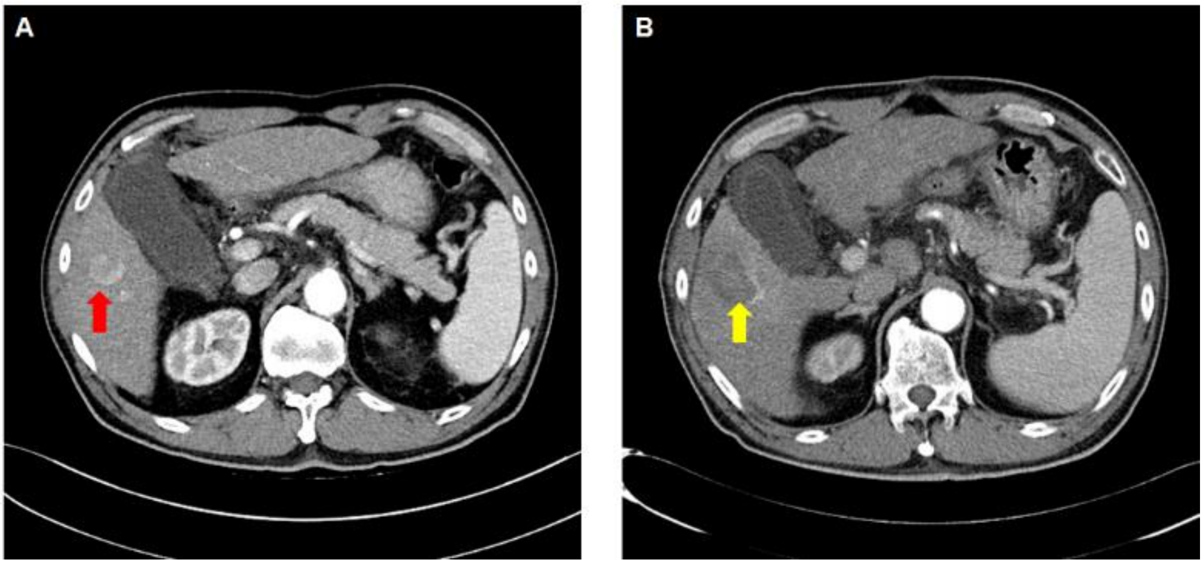

Figure S1. Representative images where the red and yellow arrows indicate HCC before RFA (A) and CR status after RFA (B), respectively. Abbreviations: HCC, hepatocellular carcinoma; RFA, radiofrequency ablation; CR, complete response.

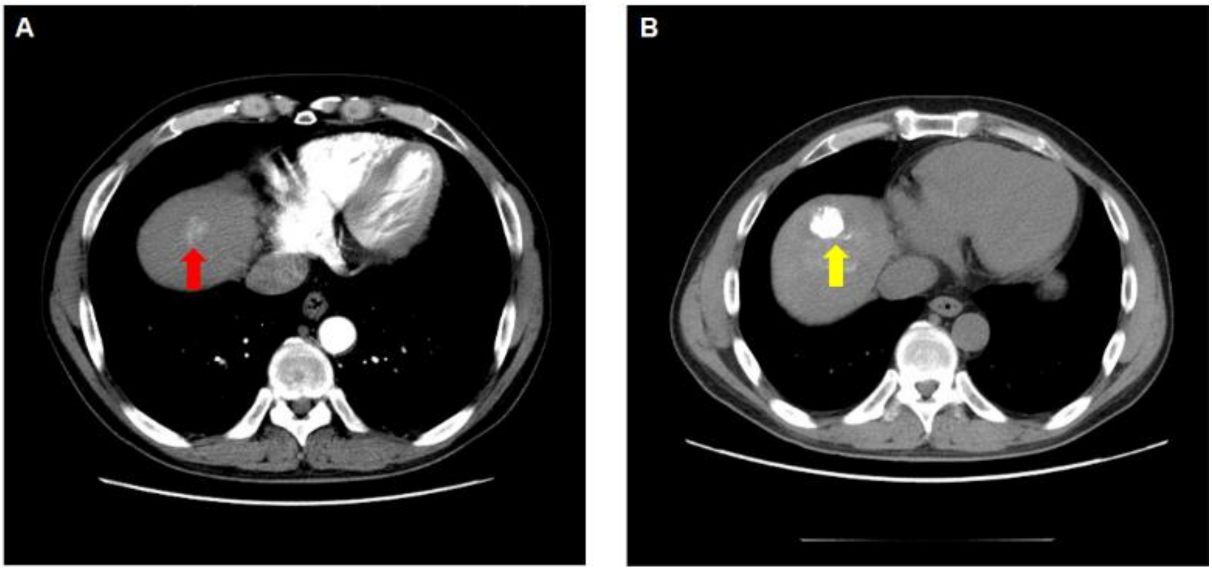

Figure S2. Representative images where the red and yellow arrows indicate HCC before TACE (A) and CR status after TACE (B), respectively. Abbreviations: HCC, hepatocellular carcinoma; TACE, trans-arterial chemoembolization; CR, complete response.

© 2020 by the authors. Licensee MDPI, Basel, Switzerland. This article is an open access article distributed under the terms and conditions of the Creative Commons Attribution (CC BY) license (http://creativecommons.org/licenses/by/4.0/).
